# Salvage-Radiation Therapy and Regional Hyperthermia for Biochemically Recurrent Prostate Cancer after Radical Prostatectomy (Results of the Planned Interim Analysis)

**DOI:** 10.3390/cancers13051133

**Published:** 2021-03-06

**Authors:** Marcus Beck, Pirus Ghadjar, Felix Mehrhof, Daniel Zips, Frank Paulsen, Daniel Wegener, Susen Burock, David Kaul, Carmen Stromberger, Jacek Nadobny, Oliver J. Ott, Rainer Fietkau, Volker Budach, Peter Wust, Arndt-Christian Müller, Sebastian Zschaeck

**Affiliations:** 1 Charité—Universitätsmedizin Berlin, Corporate Member of Freie Universität Berlin, Humboldt-Universität zu Berlin, and Berlin Institute of Health, Department of Radiation Oncology, 13353 Berlin, Germany; pirus.ghadjar@charite.de (P.G.); felix.mehrhof@charite.de (F.M.); david.kaul@charite.de (D.K.); carmen.stromberger@charite.de (C.S.); jacek.nadobny@charite.de (J.N.); volker.budach@charite.de (V.B.); peter.wust@charite.de (P.W.); sebastian.zschaeck@charite.de (S.Z.); 2 Department of Radiation Oncology, University Hospital Eberhard-Karls-University Tübingen, 72076 Tübingen, Germany; Daniel.Zips@med.uni-tuebingen.de (D.Z.); Frank.Paulsen@med.uni-tuebingen.de (F.P.); Daniel.Wegener@med.uni-tuebingen.de (D.W.); Arndt-Christian.Mueller@med.uni-tuebingen.de (A.-C.M.); 3 Charité Comprehensive Cancer Center, Charité Universitätsmedizin Berlin, 10117 Berlin, Germany; susen.burock@charite.de; 4 Department of Radiation Oncology, Universitätsklinikum Erlangen, 91054 Erlangen, Germany; oliver.ott@uk-erlangen.de (O.J.O.); rainer.fietkau@uk-erlangen.de (R.F.); 5 Berlin Institute of Health, 10117 Berlin, Germany

**Keywords:** prostate cancer, salvage radiation therapy, postoperative, hyperthermia, radiosensitization, dose-escalation

## Abstract

**Simple Summary:**

Several efforts like dose-escalated salvage radiation therapy and the use of androgen deprivation therapy aimed to improve the postoperative treatment in patients with biochemical recurrence of prostate cancer after prostatectomy. However, the oncological outcome is still not satisfactory. Hyperthermia is well-known to improve the efficacy of radiation therapy, whereas only limited data for postoperative therapy in prostate cancer are available. Thus, we conducted a prospective multicenter non-randomized Phase-II-Trial (HTProstate) investigating the implementation of combined salvage radiation therapy and regional hyperthermia in case of biochemical recurrence after prostatectomy with the aim to evaluate the safety, feasibility, and oncological outcome of this approach. The results of our planned interim analysis (n = 50) met the criteria of safety (only one patient with acute grade 3 hyperthermia-specific toxicity), showed feasibility of planned radiation and hyperthermia therapy, no significant changes in quality of life and promising short-term prostate-specific antigen response. Late toxicity and robust oncological outcome data will be reported after completion of the trial.

**Abstract:**

Efforts to improve the outcome of prostate cancer (PC) patients after radical prostatectomy (RP) include adjuvant or salvage radiation therapy (SRT), but still up to 50% of patients develop a disease progression after radiotherapy (RT). Regional hyperthermia (HT) is well-known to improve tumor sensitivity to RT in several entities. Here we report on a planned interim analysis of tolerability and feasibility after recruitment of the first 50 patients of a trial combining SRT and HT. We conducted a prospective multicenter non-randomized Phase-II-Trial (HTProstate-NCT04159051) investigating the implementation of combined moderate-dose escalated SRT (70 Gy in 35 fractions) and locoregional deep HT (7–10 HT sessions). The primary endpoints were the rate of acute genitourinary (GU), gastrointestinal (GI), and HT-related toxicities, completed HT sessions (≥7), and SRT applications per protocol (≥95% of patients). The two-step design included a planned interim analysis for acute GU-, GI- and HT-specific toxicities to ensure patients’ safety. Between November 2016 and December 2019, 52 patients entered into the trial. After 50 patients completed therapy and three months of follow-up, we performed the planned interim analysis. 10% of patients developed acute grade 2 GU and 4% grade 2 GI toxicities. No grade ≥3 GU or GI toxicities occurred. HT-specific symptoms grade 2 and 3 were observed in 4% and 2% of all patients. Thus, the pre-specified criteria for safety and continuation of recruitment were met. Moreover, ≥7 HT treatments were applicable, indicating the combination of SRT + HT to be feasible. Evaluation of early QoL showed no significant changes. With its observed low rate of GU and GI toxicities, moderate and manageable rates of HT-specific symptoms, and good feasibility, the combined SRT + HT seems to be a promising treatment approach for biochemical recurrence after RP in PC patients.

## 1. Background

Radical prostatectomy (RP) is the preferred treatment for many patients with localized prostate cancer (PC). After RP, up to 40% of patients develop a biochemical recurrence (BR), and the predominant site of failure is local [1,2]. In the case of BR salvage radiation therapy (SRT) provides a potentially curative treatment option with favorable oncological results in randomized trials [3,4,5,6]. However, up to 50% of patients develop further disease progression after SRT [7,8,9]. Consequently, several efforts aimed at an improvement in the oncological outcome of SRT. Randomized controlled trials of dose-escalation for definitive radiation therapy (RT) of PC revealed a benefit, yet limited level 1 evidence is available so far for dose-escalated SRT [10,11,12]. However, retrospective data have shown a superior biochemical relapse-free survival for this patient cohort compared to standard dose SRT [13,14,15,16]. The combination of SRT and androgen deprivation therapy (ADT) is an alternative approach to optimize the oncological outcome in BR after RP. Data of two randomized trials showed benefits. In the first trial, an advantage in overall survival (OS), cancer-specific survival (CSS), and metastasis-free survival (MFS), particularly for patients with high-risk factors, were observed, and in the second trial, an improvement in freedom of biochemical and clinical progression [17,18]. As ADT is associated with severe adverse events and a deterioration of quality of life (QoL), its risks in patients have to weigh up carefully against their benefits [19,20,21]. A considerable number of patients refuse ADT due to QoL-related concerns. Hence, additional methods to improve outcomes after SRT are warranted. The combination of RT with regional hyperthermia (HT) is another option for improved therapeutic effects [22]. Promising results in a variety of cancer types were reported [23,24]. In case of PC, published data showed an oncological benefit of additional HT combined with definitive RT in advanced PC that comes along with good tolerability and no decline of QoL [25,26,27,28,29]. However, only limited data are available for combined RT and HT in postoperative BR after RP [n = 10 in two cited reports] [25,27]. Moreover, Kok et al. published a method to estimate the additional dose benefit from combined RT and HT. The authors calculated a minimum average increase of 10 Gy equivalent dose when HT was added [30]. Thus, considering the reported improvement in outcomes by radiotherapeutic dose-escalation, HT seems also a promising option. For example, the model published by King et al. estimated an increase of relapse-free survival after SRT of 2% per each Gray of dose escalation [16]. However, dose escalation is limited by rising rates of genitourinary (GU) and gastrointestinal (GI) toxicity which affect QoL [11,15]. We hypothesize that the combination of dose-intensified RT (70 Gy) and additional radiosensitization by simultaneous HT could be a promising therapeutic option to achieve a better oncological outcome that comes along with satisfactory tolerability and without relevant deterioration of QoL. Whether this aim is achievable is subject to the investigations of the ongoing prospective phase-II HTProstate trial [31]. After reaching the planned first step of recruitment acute toxicity, feasibility and early QoL were analyzed according to protocol. The findings are presented in this publication.

## 2. Results

### 2.1. Patient Characteristics

Between November 2016 and December 2019, 52 patients were included in the HTProstate-Trial (43 patients at Charité Universitätsmedizin Berlin, Germany; nine patients at Eberhard Karls University Tübingen, Germany). Hence, after 50 out of the initial 52 patients completed the therapy and the three months follow-up, we reached the first step of the trial and performed the planned interim analysis as per protocol.

One patient withdrew informed consent before trial specific treatment and decided to perform only the SRT. Another patient received the SRT + HT treatment but he withdrew his consent after 20 min of the first HT application (claustrophobic feeling and panic attack during HT treatment) and completed SRT alone.

**Figure 1:**
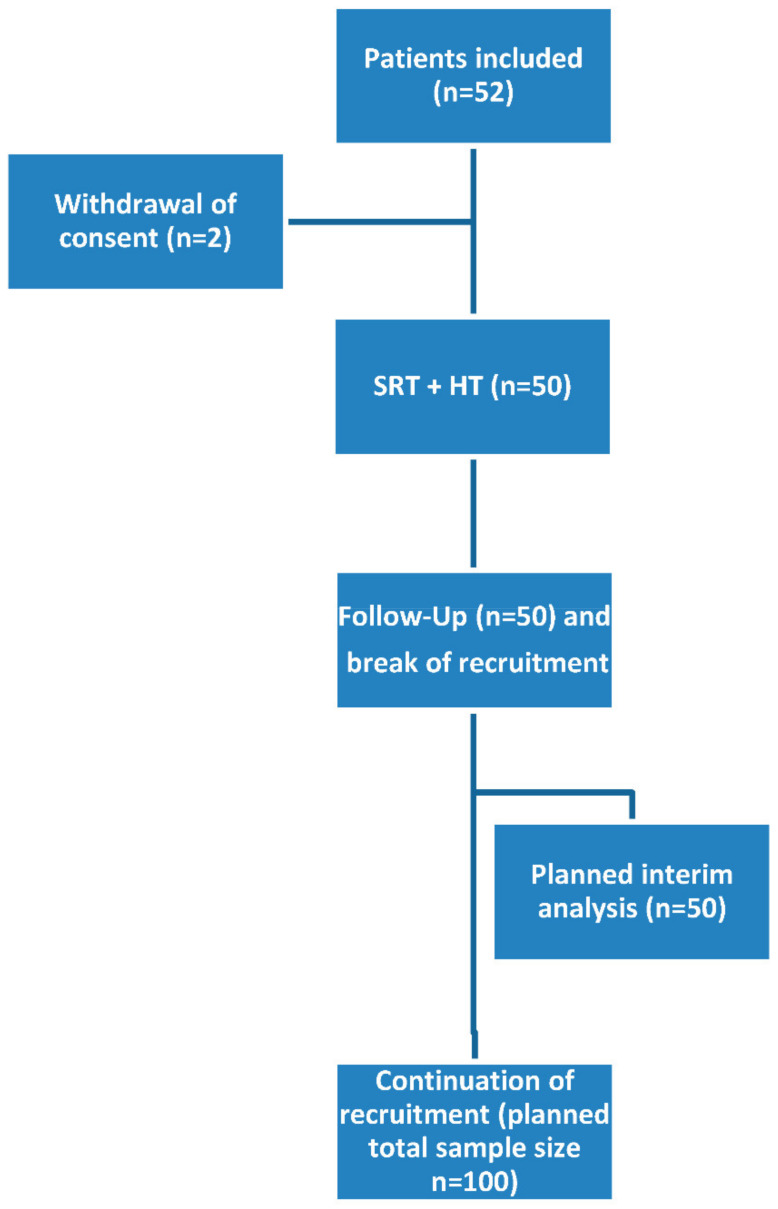
Recruitment schedule (SRT: salvage radiation therapy; HT: hyperthermia).

The safety population was consisting of 50 patients (Figure 1).

The characteristics of patients treated in this first step of the HTProstate-Trial were summarized in Table 1. The median age of patients was 65.5 years (range 51–79) with a good performance status (94% ECOG 0) and most patients had high-risk prostate cancer. The median prostate-specific antigen (PSA) at the beginning of the SRT was 0.25 ng/mL (range 0.07–0.77). Sixty-two percent of patients had a R0 resection and 72% a Gleason of 7. The median time between RP and beginning of SRT was 21.5 months (range 3–128).

### 2.2. Acute GU-, GI- and HT-Specific Toxicity

At baseline eighteen (36%) patients had grade 1 GU, three (6%) had grade 2 GU and no patients had grade 3 GU symptoms (Table 2).

Three (6%) patients experienced grade 1 GI symptoms and no grade 2 or 3 GI symptoms were detected at baseline examination (Table 3).

Evaluating the weekly toxicity screenings during therapy, we could detect a rate of 54% grade 1, 10% grade 2, and no grade 3 acute GU toxicities. At three months follow-up 38% of patients suffered from grade 1, 6% from grade 2 and none from grade 3 acute GU toxicities (Table 2).

During therapy, 24% and 4% of patients, and at three months follow-up 10% and 2% of patients developed grade 1 or grade 2 acute GI toxicities, respectively. Neither during therapy, nor at three months follow-up did any grade ≥3 GI toxicities occur (Table 3).

HT-specific symptoms grade 1 were documented in 42%, grade 2 in 2% and grade 3 in one patient (2%) during or shortly after the HT series. At three months follow-up 6% grade 1 and 4% grade 2 HT-specific symptoms were observed (Table 4).

Only one grade 3 event (grade 3 abdominal pain from HT, [CTCAE grading: severe pain, limiting self-care ADL]) occurred in one patient during the HT sessions 6 and 8. Both treatment sessions had to be interrupted (session six at minute 50 of therapeutic HT phase, session eight at minute 20 of therapeutic HT phase). The physician treated the pain both times with Metamizole (WHO °1 analgetic). This medication led to fast relief of pain and the patient tolerated the next planned HT (session 7, respectively session 9 + 10) without problems and completed overall 10 HT treatments.

Eighteen patients reported a grade 1 bolus pressure and grade 1 hot spots during HT treatment, respectively. One patient suffered from grade 1 claustrophobia while lying in the HT applicator.

Three patients developed a superficial burning grade 1 of the skin that healed fast under adequate supportive therapy. Details of the burnings: case 1: 2 cm diameter, tailbone/rima ani region, occurred at 8th HT session; case 2: 1.5 cm diameter, tailbone/rima ani region, occurred at 8th HT session; case 3: 1.5 cm diameter, tailbone/rima ani region, occurred at 7th HT session. In one case a grade 2 burning with a blister appeared in the tailbone/rima ani region at HT session number 6. After pausing one HT session and continuing the treatment in the following week another four HT treatments could be performed and the burning was already almost healed at the end of session number 10. The treated patients developed no higher than grade 2 burnings.

### 2.3. Feasibility and Safety

#### 2.3.1. The Numbers and Parameters of Applicated HT and SRT Treatments

Median number of performed HT treatments was 8.5 (range 2–10). Seven patients received <7 HT sessions. Exact number of completed treatments: 2 × HT n = 1, 5 × HT: n = 1, 6 × HT: n = 5, 7 × HT: n = 5, 8 × HT: n = 13, 9 × HT: n = 3, 10 × HT: n = 22.

The patient with only two treatments rejected further HT treatments due to a sensation of pain in the pubic skin and soft tissue region that stopped after switching the power off. A similar sensation was also reported by most members of the subgroup of patients that reported abdominal pain and hot spots (Table 4). Still, only one patient withdrew further treatment due to this pain. In all cases, this sensation was temporary and did not influence daily life.

Moreover, six patients received <7 HT treatments (one patient with five HT treatments, five patients with six HT treatments) without a medical or technical reason due to logistic and organizational issues (shortage of staff, maintenance, high patient volume) and were excluded from the HT feasibility analysis.

During HT therapies the following thermal parameters could be detected: mean temperature in the target region (Tmean_t_) = 40.2 °C (standard deviation [SD]: 0.67); T_90_ = 39.8 °C (SD: 0.65). SRT could be performed as per protocol (prescribed total dose) in 100% of the patients. The median total power used was 616 W (SD: 89), and the median water bolus temperature was 22.5 °C (SD: 1.4).

#### 2.3.2. Early Quality of Life

The patient reported QoL values (QLQ-C30 + QLQ-PR25) at baseline and three months after therapy (first follow-up) are listed in Table 5. The results show no significant worsening or improvement of QoL in the short-term observation. Notably, GU, GI and sexual parameters as represented in PRURI, PRBOW, PRSAC, DI, NV and CO-scores also showed no significant differences between baseline and first follow-up. The questionnaire submission was 90% at baseline and 66% at three months.

#### 2.3.3. Short-Term PSA-Development

**Figure 2:**
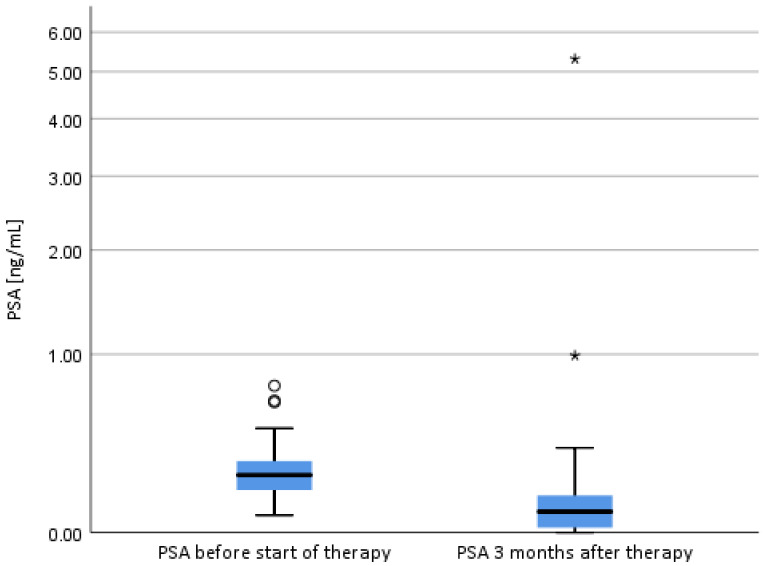
PSA values before therapy and at 3 months Follow-Up (*: 2 patients with biochemical recurrence after 3 months; PSA: prostate- specific antigen).

At three months, the PSA values decreased in most patients, aside from two patients with a detected rapid BR (Figure 2). PSA at start of RT was in median 0.25 ng/mL (0.07–0.77) and at three months of follow-up in median 0.085 ng/mL (0–5.31). The patient with the rapid BR and PSA of 5.31 ng/mL showed in a PSMA-PET-MRI one iliac lymph node- and multiple bone metastases. A PSMA-PET-CT of the second patient with rapid BR and a PSA of 0.99 ng/mL showed one iliac lymph node metastasis. In both cases no in-field recurrence was detected.

## 3. Discussion

After completing the per protocol planned safety analysis, we observed overall good tolerability (one patient [2%] with HT-related grade 3 toxicity) and feasibility of combined SRT (100% with completed full RT dose) and regional HT treatment (only 2% with <7 HT treatments) in the first 50 patients treated for biochemical failure after RP. Thus, safety criteria (<20% of grade 3+ toxicity), feasibility criteria of HT (≥95% with ≥7 HT treatments) and feasibility criteria of SRT (≥95% with completed full dose RT) as per protocol were met.

Hence, recruitment continues for the planned number of 100 patients to investigate the additional endpoints.

Our presented results show only a minor rate of toxicity with an acute grade 2 GU toxicity rate of maximum 10% and maximum 4% grade 2 GI acute and no grade 3 GU or GI radiation toxicity. Only one case of temporary grade 3 (abdominal pain) and no > grade 3 HT-specific toxicity was observed, whereas the SRT (complete dose) + HT (8 regular + 2 interrupted HT sessions) could be completed in this patient. With overall only maximum 4% of grade 2 HT-specific symptoms the HT interventions seem to be well tolerated.

Consequently, with additional HT no higher acute GU and GI toxicity in comparison to similar SRT-only schedules (for example SAKK 09/10 trial) could be detected and on the other hand, the rate of HT-specific symptoms was limited [11]. Particularly, with only one patient developing grade 3 HT-related toxicity and no grade 3 GU- or GI toxicity, this rate is considerably below the cut off as per protocol (≥20% grade 3 toxicity would indicate early termination of trial).

However, four patients (8%) suffered a burning (three grade 1 burnings, one case of grade 2 burning) that healed fast under adequate supportive care and no omission of further HT treatment was necessary. Continuous monitoring and immediate feedback (pain, heat sensation) of patients is necessary to avoid unnoticed hot spots resulting in skin burnings.

Furthermore, the thermometry with usage of temperature probes is an important part of a quality control in HT treatments, even though the placing of probes in bladder and rectum is uncomfortable for patients. As mentioned, one patient denied the HT treatment and withdrew the consent due to the required catheterization. A fact that could be optimized with advantages of noninvasive MRI-thermometry in the future.

Eighteen percent of patients stated a discomfort by bolus pressure. The used ring applicators encompass the lower abdomen and the pelvis and the distance between body and applicator antennas is bridged by a mandatory water bolus for transmission of the waves send out by the applicator antennas and superficial cooling. Moreover, one patient withdrew his consent to the trial after claustrophobic sensations at the first treatment initiation (after 20 min in the applicator).

Adaption of the body position by experienced staff could relief or solve the bolus discomfort sensation in most cases.

Most of the patients that stated abdominal pain (12%) or skin pain (10%) or hot spots (18%) described a painful dragging or pressing sensation in the lower abdominal or pubic region. Presumably, hot spots in the anterior pelvic girdle caused these symptoms. In some cases, repositioning of the patient and/or adjustment of the antenna control solved the problem. Some patients developed these sensations at every treatment until completion of therapy.

Overall, it is mandatory to guarantee a high-quality implementation of HT treatments corresponding to the current HT guidelines [32,33].

As per protocol, the feasibility of HT treatment was defined as the possibility to perform ≥7 HT sessions. Within the feasibility analysis only one patient received <7 HT treatments as a result of toxicity. In the other 6 cases the low number of treatments was not due to medical or technical problems, resulting in a feasibility of HT as defined as per protocol with 2% rate of considerable cases of <7 HT sessions. As reported organizational and logistic issues led to the minor number of HT sessions in six cases. Consequently, an optimized quality assurance program and refined organizational processes resolved these problems in the ongoing trial.

The detected thermal HT parameters showed the expected sufficient values for a heating of the prostate bed region with a mean T_90_ of 39.8 °C and a mean target temperature of >40 °C. Comparable results of pelvic thermal parameters were reached with similar standard HT settings in former published treatment/planning series [27,34,35,36].

The analysis of early QoL showed no deterioration of QLQ-C30 and prostate-specific QLQ-PR 25, thus confirming the combined SRT + HT treatment option’s good tolerability.

First short-term PSA values detected a favorable response to therapy, apart from two patients with rapid BR development, which was confirmed as out-field metastasis in both cases.

Despite numerous efforts to optimize the treatment in BR after RP in PC patients, still up to 50% of patients develop a disease progression after SRT treatment [7,8,9].

Several recently published trials reported an improved oncological outcome with a combination of SRT and ADT. In the RTOG 9601 trial SRT to a total dose of 64.8 Gy was combined with 24 months of bicalutamide and a significantly improved 12-yr overall survival of 76.3% versus 71.3% (*p* = 0.04) without ADT and a reduced cumulative incidence of metastatic disease in the experimental arm (14.5% versus 23.0%, *p* = 0.005) was observed. However, the subgroup analysis showed the significant overall survival benefit only in patients with a PSA at trial entry of ≥0.7 ng/mL [17]. Carrie et al. published the data of GETUG-16 trial investigating the combination of SRT (66 Gy) with or without six months of a luteinizing hormone–releasing hormone agonist [37]. Eighty percent of patients had a PSA of <0.5 ng/mL and the trial showed significantly superior freedom from biochemical or clinical progression in the experimental arm after five years (80% versus 62%, *p* <0.0001). Recently an update of GETUG-16 was published and described a benefit in progression-free survival after a median follow-up of 112 months with 64% for combined treatment versus 49% for SRT alone (*p* <0.0001) [18]. However, besides the mentioned benefits by combined SRT and ADT in subgroups of PC patients (for example with persisting PSA after RP, PSA of ≥0.7 at start of SRT, or patients with <0.7 ng/mL and additional risk factors like Gleason ≥8 or R0 margins in RP) we have also to realize the side effects and sometimes significant adverse events of ADT [19,38].

Currently reported results of the JCOG 0401 trial showed inferior oncological outcome in exclusive salvage ADT treatment versus SRT + ADT treatment. Thus, omission of SRT seems to be obsolete [39].

Other strategies to improve the outcome of SRT are dose-intensified RT schedules. According to results of mainly retrospective studies the dose intensification is associated with a superior biochemical control after SRT and well tolerable with slightly increased rates of acute and late GU and GI toxicity [13,14,15,16].

A recently published monocenter randomized phase-III trial of adjuvant RT and SRT in PC patients compared treatment with standard dose of 66 Gy to moderate dose-escalation with 72 Gy and could not demonstrate an improvement of biological progression-free survival between both arms after a median follow-up of 48.5 months. [12].

Likewise, data of the well-designed SRT dose-intensification phase-III trial (SAKK09/10) suggested increased late rectal toxicity without benefit in freedom from biochemical progression after dose intensified SRT with 70 Gy (Ghadjar et al., Abstract 194, ASCO Genitourinary Cancers Symposium, 2021). The previously published acute toxicity and QoL data showed good tolerability of dose-intensified treatment, however with increased genitourinary symptoms after dose-intensification [11].

The data of King et al. indicate a further oncological benefit (2% improvement of biochemical relapse free survival with each 1 Gy) even with SRT doses >70 Gy [16]. However, potentially further increase of GU and GI toxicity must be kept in mind [15].

In this context combined use of SRT and regional HT (assumed dose-benefit of minimum 10 Gy by additional HT) could lead to improved outcomes without increase of radiation toxicity [30].

Thus, in consideration of reported side effects, adverse events and deterioration of QoL in case of ADT and the rising risk of GU and GI toxicities with increasing doses in dose-intensified SRT the combination of SRT with regional HT could be an alternative approach.

However, robust oncological outcome data, late toxicity, and long-term QoL will be reported after trial completion and follow-up of the planned 100 patients.

## 4. Methods

### 4.1. Trial Design

We conducted a prospective multicenter non-randomized Phase-II-Trial (HTProstate, ClinicalTrials.gov identifier: NCT04159051) investigating the implementation of combined SRT and regional HT in PC patients with BR after RP.

### 4.2. Patients

#### Main Inclusion Criteria

Main inclusion criteria were informed consent; presence of postoperative node negative (cN0, pN0) adenocarcinoma of the prostate (RP at least 12 weeks before study inclusion. Tumor stage: pT2a-3b, R0-1 (International Union Against Cancer TNM 2009) with available Gleason score; Proven PSA progression after prostatectomy with two consecutive rises with a final PSA >0.1 ng/mL or three consecutive rises. PSA measurement at least four weeks after RP; PSA <2ng/mL at inclusion; magnetic resonance imaging (MRI) or computed tomography (CT) of the pelvis and abdomen; WHO performance status of 0–1 and age of 18–80 years.

### 4.3. Main Exclusion Criteria

Main exclusion criteria were a persistent PSA greater than 0.4 ng/mL four to 20 weeks after RP, Palpable tumor in the prostate fossa (unless no evidence of malignancy in ultrasound-guided biopsy); pelvic or abdominal lymph node enlargement larger than 1 cm in short-axis diameter, except histology is proven nonmalignant; evidence of metastatic disease or macroscopic local recurrence (MRI or CT performed within 16 weeks before randomization), an ADT or bilateral orchiectomy; Hip prosthesis; metallic implant/markers; cardiac pacemaker; active or severe comorbidities.

Further details are referred to the trial protocol publication and the entry at ClinicalTrials.gov (NCT04159051) website [31].

### 4.4. Treatment and Follow-Up Procedures

RP was not part of this trial and had to be performed at least 12 weeks before study inclusion. The mandatory MRI or CT (within 16 weeks before study inclusion) was necessary to exclude macroscopic recurrence, lymph node involvement or metastatic disease.

### 4.5. Radiation Therapy

SRT was administered >12 weeks after RP to a total dose of 70 Gy in 35 fractions (2 Gy, five days a week, over seven weeks) to the prostate bed. The use of modern RT techniques (intensity- modulated radiation therapy [IMRT] or volumetric modulated arc therapy [VMAT]) was required. In case of explicit identification of regions at risk in the prostate bed a simultaneous-integrated boost with the dose of 70 Gy (35 fractions with 2 Gy) was allowed, whereas the dose to the whole prostatic fossa had to be minimum 66.5 Gy (35 fractions with 1.9 Gy).

The prostate bed, clinical target volume (CTV) and planning target volume (PTV) were delineated according to the European Organization for Research and Treatment of Cancer (EORTC) guidelines [40]. The PTV was defined as the CTV with a 10 mm margin in all directions, except for a dorsal 8 mm margin (rectal direction). In case of daily CT-based image guided radiation therapy (IGRT) a dorsal reduction of PTV with a 5 mm margin was also allowed.

Organs at risk (OAR) included the bladder, rectum (contoured from the anus to the rectosigmoid flexure) and femoral heads. The rectal wall (RW) and bladder wall (BW) were delineated using a 5 mm internal margin. The constraints for the OAR were: RW volume receiving 60 Gy ≤50%, RW volume receiving 70 Gy ≤20%; BW volume receiving 65 Gy ≤50%; femoral heads volume for each head receiving 50 Gy ≤10%.

Dose prescription was done according to International commission on radiation units and measurements (ICRU) to the median dose of the PTV and the variation in the PTV was restricted to +7%/−5% of the prescribed dose. The 95% isodose encompassed the PTV.

A CT simulation in the supine position with an empty rectum and comfortably filled bladder was required for treatment planning.

### 4.6. Hyperthermia

The quality assurance guidelines of the European Society for Hyperthermic Oncology (ESHO) defined the use of regional HT [32,33]. The HT was administered with a BSD-2000/3D system using the Sigma-60 or Sigma-Eye applicator or a Sigma-Eye-MR applicator with the MRI-HT-hybrid-system BSD2000/3D-MRI. HT was performed twice a week (minimum 48 h interval) and as possible promptly within the first two hours after the RT (maximum up to four hours after the RT) to a total number of maximum 10 HT sessions (≥7 HT sessions were aimed). The settings were performed based on own experiences in planning and application of prostate HT to reach sufficient thermal parameters [27,34,36]. After an induction period of approximately 30 min, HT treatment was performed for 60 min with a temperature of at least 40°C (aimed 41 to max 43 °C) at the reference points. For observation of temperature at the reference points, a thermal mapping with multichannel thermometry probes or MRI-mapping temperature probes was required. The temperature probes were placed in a rectal and additional in a urinary catheter (urethra, bladder).

### 4.7. Observation of Toxicity and QoL

The acute and late GU and GI toxicity was assessed according to the National Cancer Institute Common Terminology Criteria for Adverse Events version 4.0 (CTCAE v.4.0). Events increasing over the respective baseline value during treatment and up to 3 months after completion of treatment were defined as acute toxicity events. Besides, HT-specific symptoms like pain of the skin, abdominal pain, bolus pressure, thermal hot spots, edema, burning and claustrophobia were also monitored. The acute toxicities had to be documented at the beginning of therapy, weekly during therapy and 3 months after completion of therapy. The late toxicity screening is required 6, 12, 18, 24, 30 and 36 months after end of therapy.

The QoL was measured with the EORTC QoL questionnaire (general module QLQ-C30 and prostate specific module QLQ-PR25) [41] and the Memorial Anxiety Scale for Prostate Cancer (MAX-PC) questionnaire [42] at the beginning and with QLQ-C30 and QLQ-PR25 at 3, 12, 36 and 60 months after completion of therapy.

### 4.8. Follow-Up

Additional to the toxicity and QoL assessment, follow up visits were scheduled at 3, 12, 18, 24, 30 and 36 months after termination of SRT for registration of survival status, PSA-values and clinical examination, medical imaging if required, biopsy if indicated and consecutive PC specific therapies (ADT, etc.). After 36 months the follow-up is completed.

### 4.9. End Points, Study Design and Sample Size

#### 4.9.1. Primary Endpoint

The primary endpoint is the rate of acute GU, GI (CTCAE v.4.0) and HT-related toxicities (particularly ≥grade 3) with the aim to assess the safety and feasibility of combined SRT and regional HT. The rate of completed HT sessions (≥7) and completed full dose SRT applications per protocol (both in ≥95% of patients) were defined as indicators of feasibility. The population for the safety analysis of HTProstate-Trial consists of every included patient with a minimum of one fraction of RT and at least one HT treatment and a follow-up of three months.

#### 4.9.2. Secondary Endpoints

Secondary endpoints are the late GU and GI toxicities and long term QoL (EORTC). Further, the biochemical progression-free survival (progression defined as PSA-rise >0.4 ng/mL or increasing PSA-level in case of initial PSA-values >0.4 ng/mL, time to progression measured from study inclusion to evidence of progression, patients with no progression censored at the time of the last follow-up) and clinical progression-free survival (progression defined as clinical progression, initiation of ADT or death, time to progression measured from study inclusion to time of event patients with no progression censored at the time of the last follow-up) and the time to ADT initiation (time to event measured from study inclusion to time of ADT initiation, patients with no ADT censored at the time of the last follow-up) will be analyzed as secondary endpoints.

#### 4.9.3. Study Design and Sample Size

The HTProstate trial is a multicenter, two-step non-randomized prospective Phase-II-trial. The two-step design (Simon’s design) included a first phase with 50 patients and a planned interim-analysis of acute GU-, GI- and HT-specific toxicities to ensure patients’ safety. A rate of ≥20% grade 3 toxicities was defined as an unacceptable event that would lead to a termination of the trial. Otherwise, the trial will be continued until the inclusion of 100 patients. The significance level is *p* < 0.05, with a power of 87.2%.

### 4.10. Statistical Analysis

Descriptive and additional statistical analysis of the trial data were performed with IBM SPSS Statistics 25 software (IBM, New York, NY, USA). For analysis of QoL data, a paired *t*-test was used.

## 5. Conclusions

In summary, the combined dose-intensified SRT and regional HT therapy seem to be a promising alternative treatment approach for BR after RP in PC patients. A low acute GU- and GI toxicity rate, particularly no increase of GU- and GI- symptoms than in similar SRT schemes, a moderate and manageable rate of HT-specific symptoms and good feasibility was observed. Late toxicity and oncological outcome data, as well as other defined endpoints as per protocol will be reported after the inclusion of 100 patients and appropriate follow-up.

## Figures and Tables

**Table 1:** Patients characteristics (PSA: prostate-specific antigen; RT: radiation therapy, WHO: world health organization).

Variable	(N = 50) n (%)
Resection margins	
R0	31 (62.0)
R1	19 (38.0)
Gleason score	
≤6	2 (4.0)
7	36 (72.0)
≥8	12 (24.0)
Tumor classification	
pT2a	9 (18.0)
pT2b	0
pT2c	22 (44.0)
pT3a	8 (16.0)
pT3b	11(22.0)
pT4	0
Lymphadenectomy performed	
No	1 (2.0)
Yes	49 (98.0)
Lymphnode classification	
N0	50(100.0)
pN0	49 (98.0)
cN0	1 (2.0)
PSA at start of RT (ng/mL), median range	0.25 (0.07–0.77)
Age at start of RT median (range) in years	65.5 (51–79)
Time from surgery to RT start, median (range) in months	21.5 (3–128)
WHO performance status at treatment start	
0	47 (94.0)
1	3 (6.0)

**Table 2:** Acute GU-Toxicity (GU: genitourinary; CTCAE: National Cancer Institute Common Terminology Criteria for Adverse Events version 4.0).

GU Toxicity	CTCAE Grade	Baseline Symptoms n (%)	During Treatment n (%)	Follow-Up at 3 Months n (%)
Cystitis	0	48 (96.0)	49 (98.0)	48 (96.0)
	1	1 (2.0)	1 (2.0)	1 (2.0)
	2	0	0	0
	3	0	0	0
	missing	1 (2.0)	0	1 (2.0)
Urinary	0	36 (72.0)	45(90.0)	33 (66.0)
incontinence	1	10 (20.0)	4(8.0)	13 (26.0)
	2	3 (6.0)	1(2.0)	3 (6.0)
	3	0	0	0
	missing	1 (2.0)	0	1 (2.0)
Urinary	0	46 (92.0)	50 (100.0)	46 (92.0)
retention	1	3 (6.0)	0	3 (6.0)
	2	0	0	0
	3	0	0	0
	missing	1 (2.0)	0	1(2.0)
Urinary	0	34(68.0)	20 (40.0)	33 (66.0)
frequency	1	15(30.0)	26 (52.0)	14 (28.0)
	2	1(2.0)	4 (8.0)	1 (2.0)
	3	0	0	0
	missing	0	0	2 (4.0)
Urinary	0	39 (78.0)	22 (44.0)	36 (72.0)
urgency	1	11(22.0)	25 (50.0)	12 (24.0)
	2	0	3 (6.0)	0
	3	0	0	0
	missing	0	0	2 (4.0)
Urinary	0	47 (94.0)	49 (98.0)	48 (96.0)
hemorrhage	1	0	1 (2.0)	0
	2	0	0	0
	3	0	0	0
	missing	3 (6.0)	0	2 (4.0)
Highest	0	29 (58.0)	18 (36.0)	27 (54.0)
grade of GU	1	18 (36.0)	27 (54.0)	19 (38.0)
symptoms /	2	3 (6.0)	5 (10.0)	3 (6.0)
toxicity	3	0	0	0
	missing	0	0	1 (2.0)

**Table 3:** Acute GI-Toxicity (GI: gastrointestinal; CTCAE: National Cancer Institute Common Terminology Criteria for Adverse Events version 4.0).

GI Toxicity	CTCAE Grade	Baseline Symptoms n (%)	During Treatment n (%)	Follow-Up at 3 Months n (%)
Anal or	0	49 (98.0)	46 (92.0)	47(94.0)
rectal	1	0	2 (4.0)	1 (2.0)
hemorrhage	2	0	2 (4.0)	1 (2.0)
	3	0	0	0
	missing	1 (2.0)	0	1 (2.0)
Diarrhea	0	48 (96.0)	45 (90.0)	47 (94.0)
	1	1 (2.0)	5 (10.0)	2 (4.0)
	2	0	0	0
	3	0	0	0
	missing	1 (2.0)	0	1 (2.0)
Rectal pain	0	48 (96.0)	41 (82.0)	48 (96.0)
	1	2 (4.0)	8 (16.0)	1 (2.0)
	2	0	1 (2.0)	0
	3	0	0	0
	missing	0	0	1 (2.0)
Fecal	0	50(100.0)	49 (98.0)	48 (96.0)
incontinence	1	0	1 (2.0)	1 (2.0)
	2	0	0	0
	3	0	0	0
	missing	0	0	1 (2.0)
Highest	0	47(94.0)	36 (72.0)	43 (86.0)
grade of GI	1	3 (6.0)	12 (24.0)	5 (10.0)
symptoms /	2	0	2 (4.0)	1 (2.0)
toxicity	3	0	0	0
Highest	missing	0	0	1 (2.0)

**Table 4:** Acute HT-specific symptoms.

HT Symptoms	CTCAE Grade	During Treatment n (%)	Follow-Up at 3 Months n (%)
Skin pain	0	45 (90.0)	49 (98.0)
	1	5 (10.0)	0
	2	0	0
	3	0	0
	missing	0	1 (2.0)
Abdominal pain	0	44 (88.0)	47 (94.0)
	1	5 (10.0)	1 (2.0)
	2	0	1 (2.0)
	3	1 (2.0)	0
	missing	0	1 (2.0)
Edema	0	48 (96.0)	46 (92.0)
	1	2(4.0)	2 (4.0)
	2	0	1 (2.0)
	3	0	0
	missing	0	1 (2.0)
Burn	0	46 (92.0)	49 (98.0)
	1	3 (6.0)	0
	2	1 (2.0)	0
	3	0	0
	missing	0	1 (2.0)
Claustrophobia	0	49(98.0)	#
	1	1(2.0)	#
	2	0	#
	3	0	#
	missing	0	#
Hot spots	0	41 (82)	#
	1	9 (18.0)	#
	2	0	#
	missing	0	#
Bolus pressure	0	41 (82.0)	#
	1	9 (18.0)	#
	2	0	#
	missing	0	#
Highest	0	27 (54.0)	44 (88.0)
grade of HT	1	21 (42.0)	3 (6.0)
symptoms	2	1 (2.0)	2 (4.0)
	3	1 (2.0)	0
	missing	0	1 (2.0)

# not examinated in 3 months Follow-Up (HT: hyperthermia; CTCAE: National Cancer Institute Common Terminology Criteria for Adverse Events version 4.0).

**Table 5:** Baseline and early patient reported quality of life scores (QLQ-C30 + QLQ-PR25).

QLQ-PR25	Baseline		3-Months-Follow-Up		
	Number of Respondents	Mean (sd)	Number of Respondents	Mean (sd)	*p*-Value ^#^
Symptom Scales: *					
Urinary symptoms (PRURI)	44	23.1 (16.8)	32	23.4 (16.1)	0.672
Bowel symptoms (PRBOW)	40	4.9 (6.8)	31	4.8 (9.6)	0.845
Functional Scales: **					
Sexual activity (PRSAC)	42	59.5 (31.7)	30	62.8 (28.9)	0.780
Sexual functioning (PRSFU)	20	52.3 (18.6)	15	58.0 (28.6)	0.621
**QLQ-C30**	**Baseline**		**3-Months-Follow-Up**		
	**Number of Respondents**	**Mean (sd)**	**Number of Respondents**	**Mean (sd)**	***p*** **-Value ^#^**
Symptom Scales: *					
Fatigue (FA)	45	17.5 (19.4)	33	15.3 (21.8)	0.386
Nausea and Vomiting (NV)	45	0.37 (2.5)	32	2.1 (7.0)	0.263
Pain (PA)	45	9.6 (16.9)	33	8.1 (16.7)	0.707
Dyspnoe (DY)	45	8.1 (17.6)	32	10.4 (23.1)	0.831
Insomnia (SL)	45	21.5 (24.8)	33	19.2 (26.4)	0.662
Appetite Loss (AP)	45	5.9 (17.8)	32	4.2 (11.2)	0.325
Constipation (CO)	45	2.2 (8.4)	32	2.1 (8.2)	1.0
Diarrhoea (DI)	43	7.7 (19.0)	29	10.3 (20.1)	0.17
Financial Difficulties (FI)	44	0 (0)	29	2.3 (8.6)	0.161
Functional Scales: **					
Physical Functioning (PF2)	45	93.5 (11.2)	32	92.3 (13.2)	0.946
Role Functioning (RF2)	45	89.3 (18.5)	32	83.9 (24.4)	0.347
Emotional Functioning (EF)	44	77.5 (21.6)	29	82.1 (21.0)	0.770
Cognitive Functioning (CF)	44	93.6 (12.0)	29	90.8 (14.5)	0.394
Social Functioning (SF)	44	85.6 (19.2)	29	82.2 (24.8)	0.259
Global health status (QOL):					
	44	72.9 (17.7)	29	72.7 (17.2)	0.458

[QLQ-PR25 = EORTC quality of life prostate cancer module PR25; QLQ-C30 = EORTC quality of life of cancer patients questionnaire; sd = standard deviation; QOL: quality of life; ^#^ by paired t-Test; * Range 0–100, with a positive score indicating a worsening; ** Range 0–100, with a positive score indicating an improvement].

## Data Availability

Research data are stored in an institutional repository at study coordinating center of Department of Radiation Oncology, Charité Universitätsmedizin Berlin (Berlin, Germany) and will be shared upon request.
